# A rare cutaneous manifestation of immune checkpoint inhibitor therapy

**DOI:** 10.1002/ccr3.7151

**Published:** 2023-04-02

**Authors:** John Cherneskie, Alyssa Tuan, Zachary Corey, Hyma Polimera

**Affiliations:** ^1^ Penn State College of Medicine Hershey Pennsylvania USA

**Keywords:** Immune checkpoint inhibitor, leukoclastic vasculitis

## Abstract

Leukocytoclastic vasculitis can be an uncommon and/or underreported adverse event of immune checkpoint inhibitor therapy, an established cancer treatment option. Differentiation among other cutaneous manifestations of adverse medication reactions—such as Stevens–Johnson syndrome, erythema multiforme, and drug reaction with eosinophilia and systemic symptoms (DRESS) syndrome—is crucial for guiding management.

Leukocytoclastic vasculitis can be an uncommon and/or underreported adverse event of immune checkpoint inhibitor therapy, an established cancer treatment option. Differentiation among other cutaneous manifestations of adverse medication reactions—such as Stevens–Johnson syndrome, erythema multiforme, and drug reaction with eosinophilia and systemic symptoms syndrome—is crucial for guiding management.

A 74‐year‐old man diagnosed with solitary metastatic melanoma of the brain underwent near‐total resection of the brain metastasis and adjuvant gamma knife radiosurgery. He was then started on ipilimumab–nivolimumab. Ten days after completing the first cycle of ipilimumab–nivolimumab, he presented with multiple painful and purpuric lesions extending from the feet to the groin and arms, but sparing the trunk, face, and mucosa (Figure 1). Physical examination showed bilateral lower extremity edema, hemorrhagic crusts, erosions, xerosis, and lichenification. Which is the most likely etiology of the skin lesions?

- Stevens–Johnson syndrome
- Erythema multiforme
- Leukocytoclastic vasculitis
- Drug reaction with eosinophilia and systemic symptoms (DRESS) syndrome

**FIGURE 1:**
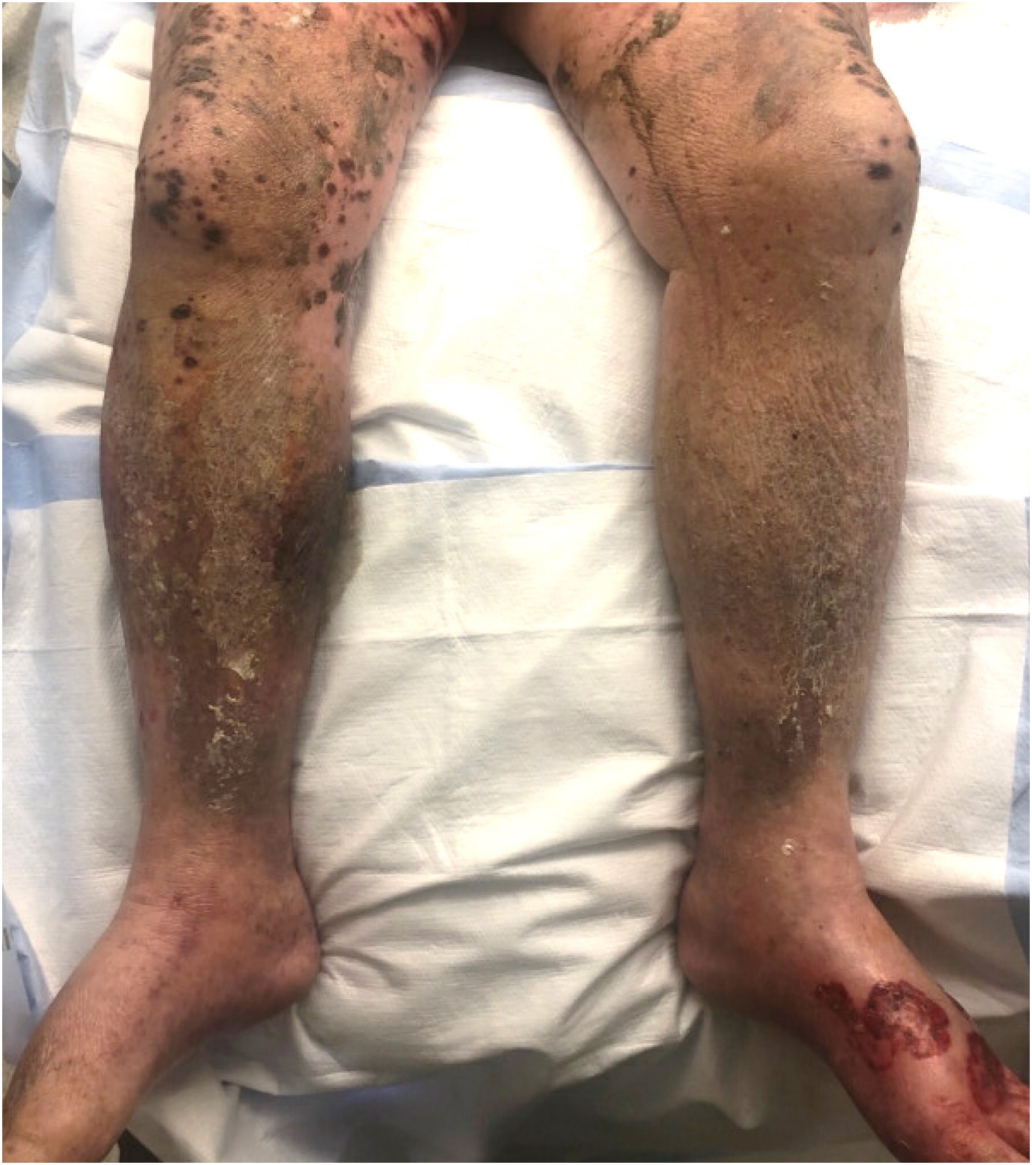
Photo representation of the purpuric lesions of the patient's bilateral lower extremities in various stages of healing.

The lesions were unlikely due to Stevens–Johnson syndrome as they spared the mucosa, trunk and face, and were not characterized largely by extensive necrosis with epidermal detachment (e.g., Nikolsky sign). The lesions were not targetoid in appearance—three concentric circles with the peripheral ring erythematous, middle zone clearer and palpable, and center erythematous and covered by a blister—as would be expected for erythema multiforme. Acral and mucosal regions are also commonly involved in erythema multiforme, which were not areas involved in this patient. Last, the patient did not have eosinophilia that might indicate DRESS syndrome, a type IV hypersensitivity reaction that is also characterized by generalized maculopapular exanthem, which was not present in this patient. The patient was diagnosed with leukocytoclastic vasculitis secondary to the immune checkpoint inhibitors. Skin biopsy revealed neutrophil‐predominant leukocytoclastic vasculitis affecting papillary and adventitial dermis consistent with IgA vasculitis, also known as Henoch–Schonlein purpura, as confirmed by immunofluorescence.

Leukocytoclastic vasculitis is a small‐vessel vasculitis in which the inflammatory infiltrate is composed of neutrophils. After degranulation, neutrophils undergo death, a process named leukocytoclasia. A review of medical literature shows most immune‐related adverse events will not present as vasculitis; it is an uncommon manifestation and/or underreported.^1^ The exact mechanism of injury is unknown but may include T‐cell overactivation, increased autoantibodies, and/or pre‐existing deficiencies in programmed cell death 1 (PD1).^2^ The patient was treated with cessation of the immune checkpoint inhibitors, high‐dose steroids followed by taper, and antibiotics for superimposed infection. The patient's skin lesions improved by the disappearance of the purpuric lesions. Guidance is lacking on how to treat this entity and most treatment data derive from case reports and small case series of patients with cutaneous leukocytoclastic vasculitis.^3^ Most case reports used systemic glucocorticoids, which can be initiated at a dose of 0.5 mg/kg per day of ideal body weight until new lesion formation ceases. Glucocorticoid‐sparing medications such as colchicine or dapsone may be trialed if there is an inadequate response to prednisone. Other trial therapies include mycophenolate mofetil, azathioprine, methotrexate, cyclosporine, hydroxychloroquine, minocycline, rituximab, chlorambucil, and intravenous immune globulin, which have shown variable success.^3^

## AUTHOR CONTRIBUTIONS

**John Cherneskie:** Conceptualization; funding acquisition; investigation; project administration; visualization; writing – original draft; writing – review and editing. **Alyssa Tuan:** Conceptualization; investigation; project administration; supervision; visualization; writing – original draft; writing – review and editing. **Zachary Corey:** Conceptualization; investigation; visualization; writing – original draft; writing – review and editing. **Hyma Polimera:** Conceptualization; investigation; project administration; supervision; visualization; writing – original draft; writing – review and editing.

## FUNDING INFORMATION

This publication will be funded via credit card by the authors and later reimbursed by the Penn State College of Medicine Internal Medicine Program in conjunction with the Office of Graduate Medical Education.

## CONFLICT OF INTEREST STATEMENT

To the best of our knowledge, no conflict of interest, financial, or otherwise exists among all authors.

## CONSENT

Written informed consent was obtained from the patient to publish this report in accordance with the journal's patient consent policy.

## Data Availability

1. The data that support the findings of this study are openly available in Current Rheumatology Reports at http://doi.org/10.1007/s11926‐019‐0828‐7, reference number 31115712.2. The data that support the findings of this study are openly available in PubMed Clin Rheumatol at http://doi.org/10.1007/s10067‐018‐4177‐0, reference number 29923081.3. The data that support the findings of this study are openly available on UpToDate at https://www.uptodate.com/contents/management‐of‐adults‐with‐idiopathic‐cutaneous‐small‐vessel‐vasculitis#H368147.
